# Caring in Ambulance Encounters With Older Patients With Complex Care Needs: A Phenomenographic Study

**DOI:** 10.1111/scs.70173

**Published:** 2026-01-02

**Authors:** Ann‐Therese Hedqvist, Mats Holmberg

**Affiliations:** ^1^ Department of Health and Caring Sciences Linnaeus University Kalmar/Växjö Sweden; ^2^ Centre of Interprofessional Collaboration within Emergency Care (CICE) Linnaeus University Växjö Sweden; ^3^ Department of Ambulance Service Region Kalmar Västervik Sweden; ^4^ Department of Ambulance Service Region Sörmland Katrineholm Sweden; ^5^ Centre for Clinical Research Sörmland Uppsala University Eskilstuna Sweden

**Keywords:** ambulance care, complex care needs, ethical decision‐making, lifeworld‐led care, nurses, older age, phenomenography, qualitative approach

## Abstract

**Background:**

Although caring is a core concept in nursing, its meaning is not uniform, and perceptions may vary across care settings. In ambulance care, encounters with older patients with complex care needs present challenges that may shape how caring is understood and practiced. Guided by a lifeworld‐led caring perspective, this study explores how caring is perceived and enacted in this specific clinical context.

**Aim:**

This study aims to describe variations in nurses' perceptions of caring in ambulance encounters with older patients with complex care needs.

**Methods:**

This qualitative study employed a phenomenographic approach to identify variations in nurses' perceptions of caring in ambulance encounters with older patients with complex needs. Semi‐structured interviews with 16 nurses in ambulance care from two regions in Sweden were analysed using a phenomenographic method.

**Ethical Considerations:**

Ethical approval was granted by the Swedish Ethical Review Authority. All participants provided informed consent to participate.

**Results:**

The outcome space comprised three qualitatively different ways in which nurses perceived caring: ‘Caring as balancing symptoms and medical care’, ‘Caring as negotiating responsibility’, and ‘Caring as responding to an ethical demand’.

**Conclusions:**

Caring in ambulance encounters with older patients with complex care needs is perceived as a multidimensional and contextually situated practice that integrates clinical, relational, and ethical dimensions. While caring was often expressed in terms of balancing symptoms and medical interventions, it also involved moral sensitivity, relational engagement, and professional responsibility. These findings contribute to a deeper understanding of caring as a lifeworld‐led and ethically grounded practice in ambulance care, with implications for education, reflective practice, and policy development.

## 1 Background

Caring is a core concept in nursing, grounded in the interpersonal relationship between nurse and patient. The essence of caring lies in attending to both health and suffering, and in recognising the person behind the patient [1]. According to Watson [2], caring can only be demonstrated and practiced interpersonally, guided by the patient's unique life circumstances, values, and experiences.

In the context of ambulance care, caring takes a distinct form. In Sweden, nurses are the caring profession with the main responsibility in ambulance services. Ambulance encounters often unfold under conditions that are both time‐pressured and unpredictable. Nurses must quickly build trust, interpret patients' needs, and make ethically informed decisions—often with limited information [3, 4, 5, 6, 7]. These caring actions are not only guided by professional knowledge but are also influenced by the nurse's own life experiences, values, and personal circumstances, which shape how situations are perceived and responded to. Ambulance encounters therefore represent an intersection between the lifeworlds of nurse and patient, where individual perspectives and experiences meet in a shared effort to alleviate suffering. Such encounters frequently involve an intricate interplay of medical, psychological, and social factors [8, 9, 10, 11, 12, 13]. Thus, ambulance care requires a delicate balance between delivering urgent medical interventions and establishing a meaningful, relational, and ethically grounded caring encounter [14, 15, 16].

At the same time, healthcare systems face growing demands due to global population aging [17]. Older adults commonly live with complex care needs resulting from multiple chronic conditions, cognitive impairments, and frailty [18, 19, 20, 21]. In emergency situations, these complexities are further amplified. Patients may present with general weakness, confusion, or behavioural changes, relating to serious underlying conditions [22]. Swedish studies show that nearly 60% of ambulance missions concern older adults (> 70 years), and many of these missions were associated with lower priority levels despite complex underlying conditions—highlighting how atypical or masked presentations represent a critical assessment challenge [8]. Acute changes in condition often trigger psychological and existential distress, including fear, anxiety, or loss of control, particularly when symptoms such as shortness of breath, pain, or delirium occur. The experience of being transported by ambulance may therefore evoke a sense of vulnerability and disorientation, demanding relational and emotional attunement from the caring nurse [10, 11, 23]. This emotional labour—enacting empathy, presence, and reassurance under pressure—is central to the nurse's caring role and may contribute to restoring a sense of safety and dignity in moments of crisis. Furthermore, the co‐occurrence of conditions such as heart failure, chronic obstructive pulmonary disease, diabetes, and cognitive decline can interact in life‐threatening ways, requiring nurses to navigate complex decision‐making while maintaining a holistic caring perspective. For nurses in ambulance care, this means navigating acute medical needs while also striving for a holistic and person‐centred understanding of each patient's situation, often in the face of polypharmacy, fragmented care systems, and limited access to medical histories [24, 25, 26, 27, 28, 29].

These challenges underscore the importance of recognising the patient's *lifeworld* as part of a caring assessment. The lifeworld refers to the surrounding world of everyday experience—our subjective reality shaped by personal history, relationships, and meaning‐making. It is within the lifeworld that health, illness, and suffering are experienced [30, 31]. A lifeworld‐led caring perspective emphasises the relational and contextual nature of care, integrating clinical, ethical, and existential dimensions. In contrast to other phenomenological approaches that seek to describe the essence of a phenomenon, a lifeworld‐led perspective focuses on how meaning is constituted in the relationship between the nurse and the patient, where understanding emerges through lived experience and intersubjectivity [32, 33]. Caring that overlooks this dimension risks being reductionistic. Older patients often present with non‐specific symptoms or subtle physiological changes rather than the ‘typical’ signs associated with acute illness [22]. This diagnostic ambiguity increases the risk of suboptimal care, for instance when serious conditions such as infection, myocardial infarction, or dehydration are misinterpreted or overlooked. Recent research further demonstrates how decision‐making for older patients in prehospital situations involves constant negotiation between medical urgency, patient needs, and contextual constraints, illustrating the ethical and relational complexity of such encounters [34, 35].

Despite the centrality of caring in nursing, there is limited research on how caring is understood and practiced in ambulance care [36, 37], particularly in relation to older patients with complex care needs. This study therefore aimed to describe variations in nurses' perceptions of caring in ambulance encounters with older patients with complex care needs.

## 2 Method

### 2.1 Design

This study employed a qualitative design with an inductive phenomenographic approach [38]. Phenomenography is based on the epistemological view that knowledge consists of descriptions of different ways of understanding a phenomenon, shaped by how individuals experience and conceptualise it [39]. This approach follows a second‐order perspective, focusing on how a phenomenon is perceived, rather than investigating its objective nature (first‐order perspective) [40]. Individual understanding is influenced by personal history, but can also be shaped by shared social constructions, leading to variations within a group [41]. Phenomenography aims to capture these variations and describe the different ways in which a phenomenon is understood within a group [40]. This approach was considered particularly suitable for the present study, as the aim was to explore the qualitatively different ways in which ambulance nurses perceive and enact caring in encounters with older patients with complex care needs. By focusing on the variation in perceptions rather than on the phenomenon itself, phenomenography enables insight into the collective understanding that shapes professional caring practice. The study followed the Consolidated Criteria for Reporting Qualitative Research (COREQ) [42].

### 2.2 Study Setting

In Sweden, the ambulance service organization is governed by the Health and Social Services Act [43] in conjunction with directives from the Swedish National Board of Health and Welfare [44]. Each of the country's 21 regions is responsible for providing ambulance care for its residents, with service provision carried out by both public and private entities. Since 2005, national regulations from the Swedish National Board of Health and Welfare have required that each ambulance be staffed with at least one registered nurse [44, 45]. Consequently, Swedish ambulance services represent a unique model internationally, as nurses hold a central role in both clinical decision‐making and the caring encounter.

In Sweden, older adults represent a substantial proportion of ambulance assignments—ranging from one‐third to more than half of all missions—and that many of these involve patients with multiple chronic conditions, cognitive impairment, or frailty [8, 46]. The increasing proportion of older persons with multimorbidity places growing demands on ambulance services, requiring not only medical expertise but also relational and ethical competence to address complex needs.

This study was conducted in two separate regions. The first region, located in northern Sweden, is served by a private provider and covers a population of approximately 270,000 people. Ambulance services are delivered from 14 ambulance stations, handling around 31,500 assignments annually. The second region, situated in southern Sweden, has a population of about 240,000 people, served by a public provider. Services are provided through 15 ambulance stations, managing approximately 38,000 assignments per year.

Both regions include a mix of urban, semi‐rural, and rural areas and face challenges such as variable travel distances, limited healthcare access in remote locations, and a growing population of older adults with complex care needs. Together, they offer a broad perspective on the realities of ambulance care across diverse geographical and organisational contexts.

### 2.3 Selection of Participants

A purposeful sampling strategy was employed to capture variations in perceptions of caring among nurses in ambulance care [47]. Inclusion required active duty and at least 2 years of professional experience as a nurse in ambulance care. After obtaining approval from the operational managers, the first author informed healthcare professionals at the ambulance stations about the study. Unit managers distributed an invitation email to eligible nurses using their professional contact addresses. Those interested in participating contacted the first author directly to receive further information and schedule an interview. No personal contact details were shared without the nurses' consent. All participants received verbal and written information and provided informed consent.

At the time of recruitment, approximately 300–350 nurses met the inclusion criteria across the two regions. The final sample included 16 nurses with a range of educational backgrounds, including both registered nurses and specialist nurses in prehospital emergency care (Table 1). The participants ranged in age from 29 to 57 years and had between 2 and 24 years of experience in ambulance care.

**TABLE 1:** Demographics of participants (*n* = 16).

	*n* (%)
Gender	
Men	8 (50)
Women	8 (50)
Age groups	
*≤* 30 years	2 (13)
31–40 years	5 (31)
41–50 years	4 (25)
≥ 51 years	5 (28)
Work experience in years	
*≤* 5	3 (19)
6–10	3 (19)
11–15	4 (25)
16–20	3 (19)
≥ 21	3 (19)
Education	
No specialist education	5 (31)
Specialist education	11 (69)

### 2.4 Data Collection

Data were collected between March 2020 and April 2021 through semi‐structured interviews, conducted either in person (*n* = 9) or via telephone (*n* = 7), depending on each participant's preference and availability. Participants could choose whether interviews took place at their workplace in a private room or off‐site in a quiet public location that ensured confidentiality. All settings allowed privacy and protected anonymity. Several participants (*n* = 11) asked to be interviewed during work hours. Due to workload constraints, some interviews were relatively brief. The interviews lasted between 20 and 65 min (median 42 min). Each interview began with the question: ‘Can you tell me about your experience of caring in an ambulance encounter with older patients with complex care needs?’ Follow‐up questions were used to encourage the participants to elaborate on and reflect upon their own perceptions of caring. The interview guide (Appendix S1) was pilot tested during the first interview. The questions were assessed to be relevant and comprehensive, and no further changes were deemed necessary. All sessions were audio‐recorded and transcribed verbatim by a professional transcriber bound by confidentiality; no artificial intelligence tools were used. The transcripts were checked and cleaned for accuracy by the first author prior to analysis.

### 2.5 Data Analysis

A phenomenographic analysis was conducted in accordance with Dahlgren's and Fallsberg's seven steps [48]. The first author performed the analysis in close collaboration with the co‐author. The process was iterative, moving back and forth between the steps to ensure depth and rigour.

The analytical work took place over approximately 4 months and involved several joint meetings, each lasting approximately 2 h, where preliminary codes and categories were discussed and refined. Consensus was reached through reflective dialogue and comparison of interpretations until agreement was achieved. To maintain analytical balance, both authors contributed equally and challenged each other's interpretations throughout the process.

First, we familiarised ourselves with the data by reading the transcripts multiple times. Then, significant statements reflecting the participants' perceptions of caring were identified and refined. These statements were compared so that we could discern variations and similarities, which allowed for grouping based on meaning. Next, the essence of each group was articulated, resulting in the labelling of categories. Lastly, categories were reviewed and contrasted to ensure they were distinct from one another [48].

To confirm the robustness of the findings and ensure that variations in perceptions were representative of the group rather than a few individuals, categories were cross‐tabulated against participants (see Table 2). The final stage involved constructing the outcome space, a visual and conceptual representation of the qualitatively different ways of experiencing the phenomenon and the relationships between the descriptive categories [38, 49]. The outcome space illustrated the hierarchical and relational structure of the identified categories, capturing the complexity and variation of the participants' perceptions.

**TABLE 2:** Representation of the three categories of descriptions across interviews, indicating the presence or absence of each category and the dominant one for each participant.

Participant	Caring as balancing symptoms and medical care	Caring as negotiating responsibility	Caring as responding to an ethical demand
1	X (dominant)	X	X
2	X (dominant)	X	
3	X (dominant)	X	
4	X	X (dominant)	
5	X (dominant)	X	
6	X (dominant)	X	
7	X	X (dominant)	
8	X	X	X (dominant)
9	X (dominant)	X	X
10	X (dominant)	X	X
11	X (dominant)	X	X
12	X	X (dominant)	
13	X	X (dominant)	X
14	X (dominant)	X	
15	X	X (dominant)	X
16	X	X (dominant)	

### 2.6 Ethical Considerations

The study obtained ethical approval from the Swedish Ethical Review Authority (Registration number 2020‐01219). The study adhered to the ethical guidelines of the Helsinki Declaration [50] ensuring compliance with requirements for information, consent, confidentiality, and data use. Informed consent was obtained from all participants prior to the interviews. The participants were assured of their confidentiality and informed of their right to withdraw from the study at any time. Only de‐identified and aggregated data were used in reporting, and no employer or healthcare organization had access to identifiable information, thereby protecting participants' anonymity.

## 3 Results

**FIGURE 1:**
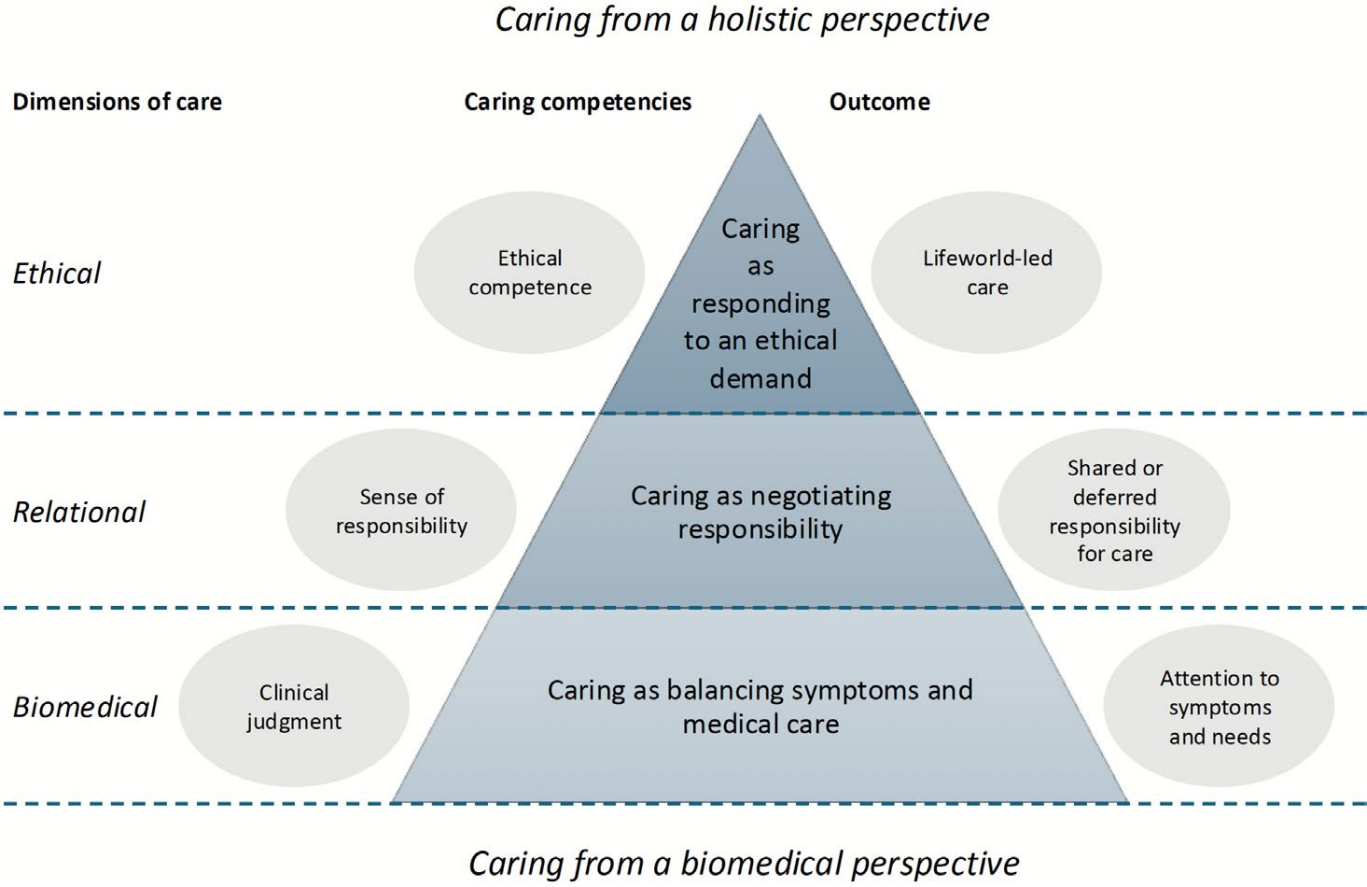
Outcome space for perceptions of caring in ambulance encounters with older patients with complex care needs.

The findings reveal three distinct categories of descriptions relating to nurses' perceptions of caring in ambulance encounters with older patients with complex care needs: ‘Caring as balancing symptoms and medical care’ (prevalent across all participants, dominant for *n* = 9), ‘Caring as negotiating responsibility’ (prevalent across all participants, dominant for *n* = 6), and ‘Caring as responding to an ethical demand’ (less consistently represented, dominant for *n* = 1) (see Table 2). These categories represent variations in how nurses perceive caring across clinical, relational, and ethical dimensions (see Table 3). The outcome space (Figure 1) illustrates the potential relationships between these categories, suggesting a hierarchical structure through which nurses navigate caring in practice.

**TABLE 3:** Categories of description, perceptions of caring, and example quotations relating to ambulance encounters with older patients with complex care needs.

Categories of descriptions	Perceptions of caring	Example quotations
Caring as balancing symptoms and medical care	Caring in the encounter with older patients with complex care needs is perceived as… Providing swift and competent medical interventions to address acute symptoms. Managing diverse and complex cases in geographically dispersed and resource‐limited settings. Applying both formal knowledge and practical expertise to ensure safe and effective care. Balancing immediate medical needs with awareness of long‐term health considerations. Using clinical judgement to assess underlying conditions beyond the obvious symptoms.	Cognitive issues are often seen in this patient group, combined with various complex somatic diagnoses, as well as hearing loss and other problems. (P5) Usually, a structured assessment will reveal the issues, but it takes longer and isn't always successful. For example, we responded to a call about a patient with digestive problems, which turned out to be high blood sugar and ketoacidosis. It's important not to miss anything and use all available tools. (P7)
Caring as negotiating responsibility	Caring in the encounter with older patients with complex care needs is perceived as… Determining the right level of care while ensuring patient safety and continuity. Bridging gaps in the healthcare system by facilitating collaboration and care transitions. Working in partnership with other healthcare providers and family members to share responsibility. Navigating professional roles and limitations while advocating for recognition of patient needs.	There's an effort to create synergy and cover for each other and coordinate things. However, there are times when the ambulance is called for situations that a municipal nurse could have assessed without needing an ambulance. (P10) You feel that the physicians have great trust in our assessments and even ask: ‘What do you think?’ It creates a very good dialogue. (P11)
Caring as responding to an ethical demand	Caring in the encounter with older patients with complex care needs is perceived as… Recognising and integrating the patient's lived experiences, values, and social context. Ensuring dignity, comfort, and person‐centred care in every interaction. Advocating for those unable to speak for themselves. Building trust and fostering meaningful connections to enhance continuity of care. Prioritising quality of life and ethical considerations beyond medical interventions.	You chose the healthcare profession for a reason—to put the patient first. Many times, patients can't advocate for themselves, and older patients often feel subordinate. (P10) It's better than taking the patient to a crowded emergency room where they lie on a hard stretcher for hours and are then sent home in a taxi at three in the morning. It's not dignified at all. (P13)

### 3.1 Caring as Balancing Symptoms and Medical Care

Nurses perceived caring as identifying, assessing, and responding to acute symptoms. This view was dominant across participants and represents a form of caring grounded in clinical competence and medical decision‐making. Caring was perceived as providing safe, efficient, and medically sound interventions. One nurse illustrated this by describing the complexity of interpreting symptoms in aging bodies and the need to adapt assessments accordingly:

They may have several illnesses that interact, with various aggravating diagnoses. Some medications can hide symptoms, or the symptoms could be side effects … or just something lagging in the aged body. Physiological responses are different in the elderly, and you rely heavily on your own experience and clinical judgment. (Participant 2)

For many nurses, caring was perceived as a dynamic form of reasoning, where the nurse continuously revised their understanding of the patient's condition.

It can be more like detective work. With a young, healthy patient, it's often a more linear process, whereas with an older, frail patient with multiple illnesses, it's a more dynamic handling. You must be prepared for that. Or be receptive to it. (Participant 6)

Contextual factors, such as geography and resources, also shaped how caring was enacted. Nurses working in rural areas emphasised the burden of making autonomous decisions when professional support was distant and transport times were prolonged.

Resource‐wise and distance‐wise, there are significant challenges. Sometimes you're hours away from the nearest hospital, with no backup. You have enormous demands on yourself to make the right decisions, you carry that responsibility alone. (Participant 11)

Although the primary focus was on medical stabilisation, nurses perceived that caring required more than technical skills. It also involved balancing immediate clinical action with an awareness of the patient's broader health context and vulnerabilities.

### 3.2 Caring as Negotiating Responsibility

Caring was also perceived as an act of negotiation—deciding what healthcare provider is best suited to meet the patient's needs and ensuring that older patients with complex care needs are not lost in care transitions. Nurses in ambulance care balance shared responsibility with deferring to other healthcare providers, especially when long‐term support, beyond emergency care, is needed.

Caring involves making judgements about when to initiate direct interventions and when to facilitate care through others. Nurses often assume a coordinating role, stepping in to fill gaps when other providers are unavailable or to bridge communication between care providers and family members. One nurse described collaborating with home care services and a family member to manage a situation in a patient's home and avoid unnecessary hospitalisation:

The home care service called us because she was more tired than usual and couldn't take her medicine … Sometimes you need to crush the pills or mix them with applesauce, which I found in her fridge. We helped change her dirty shirt and got her more comfortable in bed. After talking to her son, we realized she was her usual self. We decided it made more sense for them to visit the health care centre the next day rather than going to the emergency department just to be sent back home again. (Participant 15)

In such encounters, caring is perceived to extend beyond acute medical action and becomes embedded in the coordination of practical and relational dimensions of care. Nurses assess their patients' needs in context and act to support continuity, safety, and comfort—often in collaboration with others. However, the participants highlighted the limits of their role and the systemic challenges that come with providing care for a population whose needs extend beyond emergency medicine.

I don't feel like we lack an instrument for this patient group … but we are the wrong instrument. (Participant 6)

Although they felt confident in handling immediate concerns, they also expressed that other professionals—such as general practitioners or municipal nurses with long‐term relationships to patients—might be better equipped to provide sustained support:

Most of our patients are elderly and multimorbid, and while we can handle the assignment well, others are better suited for long‐term care … a municipal nurse or GP who knows the patients is actually much better at managing them. We manage because we see so many of them, but there are definitely others who are more adept at handling these cases. (Participant 4)

Thus, caring is marked by the nurse's ability to recognise when their involvement is beneficial and when responsibility should be transferred. It involves actively safeguarding the patient's trajectory by making considered decisions about when to act, when to refer or defer to another care level, and how to advocate for the right care at the right time.

### 3.3 Caring as Responding to an Ethical Demand

Lastly, caring was perceived as a deeply ethical and relational practice, grounded in attentiveness to the older patient's unique lifeworld. Nurses perceived a responsibility not only to treat acute symptoms but also to uphold the patient's dignity, preferences, and values in moments of vulnerability. This perspective reflects a holistic approach in which clinical decisions are embedded in a broader understanding of the patient's lived experiences and social context. Caring was perceived as creating space for presence, person‐centredness, and relational engagement. Nurses emphasised their ethical obligation to be fully present, advocate for those with limited capacity to voice their needs, and foster a sense of continuity and trust:

Especially with older patients, it is extra important to go above and beyond, to show that you are there and care about them, rather than doing everything else perfectly. In the ambulance, we have one patient at a time […] and I can give them all my time. I am only focused on them […] For most of us, person‐centred care comes naturally. You listen, you talk about small everyday things to make them feel safe. It's not always about doing more medically, but about being present and showing that they matter. (Participant 9)

This ethical commitment often emerged through sensitivity to the patient's lifeworld. Nurses highlighted how entering the patient's home provided vital contextual cues that shaped clinical judgement and relational care:

When you come to the patient's home, the patient really becomes a person for us. You quickly get a picture of the patient and their home and how they manage at home […] One of us can focus on the patient while the other goes around the apartment gathering information, talking to relatives. (Participant 8)

Caring also involved navigating ethically complex situations, such as end‐of‐life care, where nurses balanced clinical judgement with human presence and compassion. In these moments, acting in the patient's best interest sometimes meant withholding life‐prolonging interventions that were deemed non‐beneficial or undignified. Nurses described guiding families through emotionally charged situations while maintaining a focus on the patient's integrity and well‐being:

He had severe kidney disease, advanced heart failure… When I felt his pulse, I could tell it was fading. Starting CPR would have been undignified, as his organs wouldn't sustain him. The family was upset that he hadn't received more care, and that he had been given morphine. Given his severe condition and the medications he had received, I realized there had likely been a palliative decision made that hadn't been communicated to them. We had to help the family understand, guiding them to what I believed was the most dignified choice. The alternative would have been a rushed CPR attempt, resulting in a more traumatic end. Instead, we encouraged them to stay by his side, to hold his hand. In that moment, we could make it a good farewell—a peaceful time together rather than a chaotic struggle. We stayed with them and he passed away while we were there. (Participant 1)

Ultimately, caring is perceived as a moral and relational endeavour. It is through attentiveness, advocacy, and presence that nurses realise the ambition to safeguard the personhood of the older patient—particularly in moments when vulnerability is heightened and medical action alone is insufficient. Caring is then understood as a response to an ethical demand: to see and treat the patient as a human being with history, meaning, and worth.

### 3.4 Outcome Space

The outcome space illustrates a hierarchical and relational structure of nurses' perceptions of caring in ambulance encounters with older patients with complex care needs. It consists of three interrelated categories of description, organised along three dynamic dimensions of care: biomedical, relational, and ethical. These dimensions are linked to specific caring competencies and associated outcomes (see Figure 1). These dimensions are not discrete but mutually influential, each shaping and sustaining the others through ongoing interaction in practice.

At the base of the hierarchy is ‘Caring as balancing symptoms and medical care’, which was the perception most commonly expressed across all participants. This view reflects a biomedical approach to caring, focused on clinical judgement and attention to symptoms and needs.

The middle layer, ‘Caring as negotiating responsibility’, emphasises relational aspects of care, where responsibility is shared or deferred across professional and organisational boundaries. This category highlights a sense of responsibility as a central competence in navigating complex care transitions.

At the top of the hierarchy is ‘Caring as responding to an ethical demand’, a less frequently expressed but more comprehensive and morally grounded understanding of caring. Here, ethical competence is essential, and caring involves responding to the patient's unique situation and values, aiming for lifeworld‐led care.

Overall, the outcome space depicts caring as a movement between clinical, relational, and ethical dimensions rather than a linear progression. Each level enriches the others through an ongoing, reciprocal process of relational understanding, decision‐making, and moral responsiveness. Caring in ambulance encounters may move from task‐oriented symptom management to ethically informed, holistic care, depending on the nurse's perception, the situation, and contextual conditions.

## 4 Discussion

This study identified three distinct yet interconnected categories of perceptions of caring in ambulance encounters with older patients with complex care needs: *balancing symptoms and medical care*, *negotiating responsibility*, and *responding to an ethical demand*. The outcome space illustrates how caring may begin from a biomedical foundation and evolve into more relational, contextual, and ethically grounded practices—progressing from clinical judgement, through a sense of responsibility, toward ethical competence.

These perceptions of caring also shape how decisions are made in practice. Each category corresponds to a distinct mode of decision‐making: in *balancing symptoms and medical care*, decisions are grounded in the patient's immediate physiological status and clinical urgency; in *negotiating responsibility*, decisions are shaped by considerations of role boundaries and systemic handovers; and in *responding to an ethical demand*, decisions are guided by moral reflection and concern for what is right or good in a given context. Thus, perceptions of caring are closely linked to the reasoning that guides nurses' actions, ranging from ‘what needs to be done now’ to ‘who is best suited to continue care’ and, ultimately, ‘what matters most to this person’.

Our findings can be meaningfully interpreted using the EXPAND model [51] as a theoretical framework for lifeworld‐led ambulance care. The EXPAND model, developed by Holmberg [51], outlines three interlinked phases—*primary understanding*, *structural explanation*, and *secondary understanding*—to explain and understand the patient's illness or injury as intertwined with the lifeworld. The initial biomedical focus on symptoms in our findings corresponds to *primary understanding*, where nurses apply intuitive and basic clinical reasoning to manage acute conditions. As nurses engage more deeply with a patient's lifeworld, caring extends toward *structural explanation*—understanding the patient's situation within broader social and organisational contexts—and, ultimately, *secondary understanding*—which integrates clinical insight with the patient's lived experience and values. Our outcome space complements the EXPAND model [51] by illustrating how nurses' perceptions of caring span across biomedical, relational, and ethical dimensions, and how caring may evolve dynamically throughout the encounter. In doing so, it highlights the potential for progression toward more holistic, lifeworld‐led care—and the need for professional support and education to facilitate that movement.

The importance of clinical judgement was particularly evident in the first category, *balancing symptoms and medical care*, describing how nurses managed caring for complex and atypical symptoms in older patients. These situations often required a form of detective‐like reasoning, grounded in clinical experience and tacit knowledge. Although this reflects the professional ideal of evidence‐informed practice [37], it also highlights the need to move beyond linear decision‐making based solely on protocols. Previous research shows that nurses in ambulance care rely on various modes of reasoning—such as intuitive, analytical, and narrative reasoning—and often shift between them based on contextual cues and the available information [52]. Several nurses expressed concern that standardised routines could silence the patient's voice, crowding out questions like ‘How does this affect your life?’, which are central to person‐centred care [30, 53]. This tension—between procedural efficiency and human understanding—is also addressed in the EXPAND model's call to integrate clinical assessments with insights from the patient's lifeworld [51].

The second category, *caring as negotiating responsibility*, reflects how nurses navigate unclear professional and organisational boundaries—a process that aligns with the structural explanation phase of the EXPAND model [51]. Systemic gaps, such as insufficient collaboration between ambulance services, primary care, and municipal services, often left nurses feeling unsupported and responsible beyond their formal role. The expression of being ‘the wrong instrument’ for these patients illustrates a mismatch between the needs encountered and the resources available. In the absence of formal structures for shared decision‐making, nurses described engaging in ad hoc coordination—sometimes with family, sometimes with home care personnel—to secure safe, non‐hospital‐based alternatives. These findings underscore a persistent challenge in care transitions: the lack of clarity around who holds responsibility for older patients with complex needs [54]. Strengthening interprofessional collaboration and establishing defined pathways for continuity of care are essential to prevent care from becoming fragmented or not being performed. In particular, enhanced collaboration with home care and primary care is vital to support nurses in ambulance care in providing context‐sensitive care [29].

The third category, *responding to an ethical demand*, represents the holistic and deeply relational approach to caring. Here, nurses strive to be attuned to the patient as a whole person embedded in a social and existential context [2, 14, 55]. Relational presence—expressed through listening, comforting gestures, and respectful dialogue with patients and relatives—was described as integral to ethical action rather than separate from it. Nurses described how entering the patient's home made the person ‘visible’ in a new way, enabling care that aligned with individual preferences and values. The patient's home, family interactions, and personal narrative became central to the assessment and care process. This approach echoes the *secondary understanding* phase in the EXPAND model [51] and aligns with theories of ethical caring as a practice grounded in attentiveness, moral reflection, emotional responsiveness, and contextual sensitivity [14, 56]. Nurses described making difficult decisions—such as withholding futile medical interventions—not only based on clinical prognosis but with a deep commitment to preserving the patient's dignity and honouring their presumed or expressed wishes [11]. These findings resonate with international research emphasising the emotional and moral dimensions of nursing in acute settings, where moral emotions, empathy, professional values, and ethical training are shown to influence ethical awareness and decision‐making in emergency care [57, 58]. Ethical competence, in this sense, involved not only knowing *what* to do but discerning *how* to act in a morally attuned, compassionate, and person‐centred manner [37, 56].

Although this ethically grounded perception of caring was clearly articulated by some participants, it was not consistently expressed across the sample. This relative absence does not necessarily indicate a lack of moral awareness, but rather reflects the contextual and organisational realities that shape how caring can be enacted in ambulance practice [10, 12, 34, 59, 60]. Time pressure, task‐oriented routines, and expectations of rapid medical intervention may limit opportunities for reflection and relational engagement, thereby constraining the expression of ethical and lifeworld‐led caring in action. In this sense, the variation observed across participants can be understood as a manifestation of the tension between organisational efficiency and professional moral agency. These findings highlight the importance of creating structural and educational conditions that enable nurses to reflect ethically in practice and to sustain relational and compassionate forms of caring even in time‐critical contexts.

At the same time, a troubling finding was the perception among some nurses that older patients with long‐term or complex care needs did not fall within the remit of ambulance care. These patients were seen as being outside the scope of emergency services—either because their needs were chronic rather than acute, or because they required forms of support that the ambulance service was not equipped to provide. Although this perception is understandable considering structural constraints, it risks further splintering an already fragmented system in which vulnerable patients may fall through the cracks [24, 54, 61, 62]. At the same time, these findings highlight that while ambulance care is often initiated as an emergency response, caring extends beyond the immediate medical event. It unfolds as a relational and ethical practice that bridges care gaps and creates continuity in moments of vulnerability [12, 63, 64, 65]. Rather than suggesting a contradiction, this illustrates how caring in ambulance encounters is layered and dynamic—rooted in clinical action yet sustained through relational engagement and moral responsiveness. In this way, the outcome space captures caring not as a linear progression from medical to moral, but as an interplay between biomedical precision, relational understanding, and ethical reflection. Caring, in this broader sense, involves not just technical competence but also a moral orientation toward the patient's needs—regardless of whether those needs align with institutional logics or predefined categories [9, 10, 12, 66].

This study demonstrates that caring in ambulance encounters is not a static task, but a dynamic, situated practice. Although guidelines and protocols provide structure, they cannot replace the relational and contextual judgements that lifeworld‐led care requires [9]. A heavy reliance on vital signs and standard algorithms risks marginalising patients' narratives and reducing caring to procedural correctness. As our outcome space illustrates, when caring remains fixed in a biomedical frame, it risks overlooking the person behind the symptoms. When nurses utilise clinical, relational, and ethical competence, they may adapt their care approach to the patient's situation and the systemic realities in which they operate. The outcome space, in tandem with the EXPAND model, reveals both the possibilities and constraints of lifeworld‐led care in ambulance care. Supporting nurses in developing ethical competence, promoting interorganizational collaboration, and creating space for the patient's lifeworld within ambulance care are key steps toward more person‐centred care for older patients with complex needs.

### 4.1 Strengths and Limitations

This study offers insights into the perceptions of caring in ambulance encounters. One strength lies in the use of phenomenography, which is well‐suited to capture variations in how a phenomenon is experienced or conceptualised. Rather than seeking a single truth, the approach aims to identify a range of qualitatively different ways of understanding caring [67], which can inform education, practice, and policy. However, several limitations should be noted. As with any qualitative method, the findings are context‐dependent. Phenomenography focuses on subjective viewpoints [47], which means that results reflect how caring is perceived rather than how it is enacted in practice.

To strengthen credibility [68], reflexive strategies were employed throughout the research process [69], including ongoing discussions within the research team to minimise bias. Semi‐structured interviews allowed for in‐depth exploration, although the depth of responses was influenced by participants' articulation skills and the interviewer's probing. Some interviews were brief due to operational constraints during the COVID‐19 pandemic. The participants' clinical experience and familiarity with concise communication helped mitigate this limitation [70]. Importantly, although the study was conducted during the pandemic, the analysis did not indicate that the nurses' perceptions of caring were shaped by temporary or COVID‐specific conditions. Thus, the findings are considered to reflect broader professional perceptions, rather than transient experiences.

Lastly, given the traditional medical orientation of ambulance care, we find it plausible that similar perceptions of caring could be present in other emergency care contexts, such as emergency departments or urgent care services. This shared professional culture may enhance the transferability of the findings beyond the specific setting studied.

## 5 Clinical Implications and Further Research

Enhancing nurses' ethical competence is essential for strengthening lifeworld‐led care. These competencies should be prioritised in both initial education and ongoing professional development. Strengthening such skills would better equip nurses to navigate the challenges of balancing acute medical demands with relational and ethical responsibilities in time‐pressured and resource‐limited environments. Future research should explore how educational interventions and organisational conditions can support the development and application of ethical competence and lifeworld‐led care in the ambulance care context.

Beyond individual competence, the findings also highlight the need for cross‐sector education and policy initiatives that promote mutual understanding between ambulance and municipal nurses. Cross‐boundary learning—through joint case discussions, interprofessional workshops, or short‐term placements—could enhance insight into each other's scope of practice, constraints, and caring approaches. Such reciprocal learning could improve coordination, reduce unnecessary ambulance transfers, and strengthen person‐centred decision‐making for older adults with complex or palliative care needs.

At a policy level, sustainable collaboration requires organisational support and targeted funding for interprofessional education and shared practice development. Initiatives that integrate municipal and ambulance care within communities of practice could strengthen continuity, foster ethical reflection across care levels, and ultimately improve the quality and safety of care for vulnerable patient groups.

Future research should explore how educational interventions, organisational conditions, and cross‐sector learning structures can be implemented and evaluated to support the development and application of ethical competence and lifeworld‐led care in the ambulance context, and how policy frameworks might best sustain these initiatives over time.

## 6 Conclusions

This study identified three interrelated ways in which nurses perceive caring in ambulance encounters with older patients with complex care needs: balancing symptoms and medical care, negotiating responsibility, and responding to an ethical demand. The most frequently dominant perception was a focus on biomedical assessment and symptom relief. This indicates that caring in ambulance care often begins from a medical perspective but may expand into more relational and ethically grounded practices.

The findings show the need for a holistic, lifeworld‐led approach to care that integrates rapid clinical interventions with collaboration, ethical reflection, and relational engagement across care boundaries. Strengthening nurses' ethical competence—alongside clinical reasoning—is essential to ensure that the dignity, values, and overall well‐being of older patients are prioritised alongside their medical needs.

## Author Contributions

A.‐T.H. and M.H. jointly designed the study. All interviews were conducted by A.‐T.H. The analysis was led by A.‐T.H. in close collaboration with M.H. A.‐T.H. drafted the manuscript, and both authors critically reviewed and approved the final version.

## Funding

This study was supported by the Department of Research in Region Kalmar County. The funder had no involvement in the study design, data collection, analysis, interpretation, or presentation of the findings.

## Ethics Statement

The study obtained ethical approval by the Swedish Ethical Review Authority (Registration number 2020‐01219).

## Conflicts of Interest

The authors declare no conflicts of interest.

## Supporting information


**Appendix S1:** scs70173‐sup‐0001‐AppendixS1.docx.

**Appendix S2:** scs70173‐sup‐0002‐AppendixS2.docx.

## Data Availability

The data that support the findings of this study are available on reasonable request from the corresponding author.
