# Supplementary material for: Caring in Ambulance Encounters With Older Patients With Complex Care Needs: A Phenomenographic Study
Source: Scand J Caring Sci. 2026 Jan 2;40(1):e70173. doi: 10.1111/scs.70173 (PMC12757976; doi:10.1111/scs.70173)
Supplement: Supplementary file 1 — Appendix S1: scs70173‐sup‐0001‐AppendixS1.docx. [file SCS-40-0-s001.docx]

# Supplemental material 1

# Interview guide (translated from Swedish)

## Introduction to the interview:

In this interview, we are interested in your perceptions and experiences of caring in the encounter with older patients with complex care needs in the ambulance context. When we talk about older patients with complex care needs, we refer to individuals who often live with multiple chronic conditions, cognitive impairments, frailty, and overlapping social or existential challenges.

## Main question:

- Can you tell me about your experience of caring in an ambulance encounter with older patients with complex care needs?

## Follow-up prompts to deepen the narrative:

- How do you understand the concept of caring in the encounter in your role as a nurse in ambulance care?
- What does caring mean to you in these situations?
- What are the main challenges or dilemmas you encounter in caring in the encounter with these patients?
- How do you assess and respond to the needs of patients who may have both medical and social or existential issues?
- What role does person-centered care play in caring in the encounter with these patients?
- Can you recall a situation that you feel illustrates good caring in an encounter with an older patient with complex care needs?
- Can you recall a situation where caring was difficult to achieve in such an encounter?

## Closing question:

- Is there anything else you would like to add or reflect on regarding caring in the encounter with older patients with complex needs in ambulance care?
