# Supplementary material for: Caring in Ambulance Encounters With Older Patients With Complex Care Needs: A Phenomenographic Study
Source: Scand J Caring Sci. 2026 Jan 2;40(1):e70173. doi: 10.1111/scs.70173 (PMC12757976; doi:10.1111/scs.70173)
Supplement: Supplementary file 2 — Appendix S2: scs70173‐sup‐0002‐AppendixS2.docx. [file SCS-40-0-s002.docx]

# COREQ Checklist

Consolidated criteria for reporting qualitative research (COREQ): 32-item checklist.

| Item | Guide Questions/Description | Reported on |
| --- | --- | --- |
| Domain 1: Research team and reflexivity |  |  |
| 1. Interviewer/facilitator | Which author conducted the interview? | First author (ATH) |
| 2. Credentials | What were the researcher's credentials? | PhD, specialist nurse in ambulance care (ATH and MH) |
| 3. Occupation | What was their occupation at the time of the study? | PhD student and specialist nurse in ambulance care (ATH), PhD, specialist nurse in ambulance care (MH) |
| 4. Gender | Was the researcher male or female? | First author was female (ATH), last author male (MH). |
| 5. Experience and training | What experience or training did the researcher have? | Clinical experience in ambulance care, PhD with training and experience in qualitative methods |
| 6. Relationship established | Was a relationship established prior to study commencement? | Some participants were familiar with the first author due to working in the same region |
| 7. Participant knowledge of the interviewer | What did the participants know about the researcher? | They were informed about her role as a PhD student and background in ambulance care |
| 8. Interviewer characteristics | What characteristics were reported about the interviewer/facilitator? | PhD student with background in ambulance care |
| Domain 2: Study design |  |  |
| 9. Methodological orientation and theory | What methodological orientation was stated? | Phenomenography, following Dahlgren and Fallsberg |
| 10. Sampling | How were participants selected? | Purposeful sampling via contact through unit managers |
| 11. Method of approach | How were participants approached? | Initially via their manager, followed by direct contact from ATH |
| 12. Sample size | How many participants were in the study? | 16 registered nurses |
| 13. Non-participation | How many refused or dropped out? | None |
| 14. Setting of data collection | Where was the data collected? | Interviews were conducted either in person or via telephone |
| 15. Presence of non-participants | Was anyone else present besides the participants? | No |
| 16. Description of sample | What are the important characteristics of the sample? | Nurses with experience in ambulance care, from both public and private regions |
| Domain 3: Data collection |  |  |
| 17. Interview guide | Were questions, prompts, guides provided? | Yes, a semi-structured interview guide was used |
| 18. Repeat interviews | Were repeat interviews carried out? | No |
| 19. Audio/visual recording | Did the research use audio or visual recording? | Yes, audio-recordings |
| 20. Field notes | Were field notes made during and/or after the interview? | Yes |
| 21. Duration | What was the duration of the interviews? | 20 to 65 minutes |
| 22. Data saturation | Was data saturation discussed? | Not applicable to phenomenography |
| 23. Transcripts returned | Were transcripts returned to participants? | No |
| Domain 4: Analysis and findings |  |  |
| 24. Number of data coders | How many data coders were involved? | Two (ATH and MH) |
| 25. Description of the coding tree | Did authors provide a description of the coding tree? | Described through categories of description and outcome space |
| 26. Derivation of themes | Were themes identified in advance or derived from the data? | Inductively derived from the data |
| 27. Software | What software, if applicable, was used to manage the data? | Microsoft Word |
| 28. Participant checking | Did participants provide feedback on the findings? | No |
| 29. Quotations presented | Were participant quotations presented? | Yes, with anonymous codes |
| 30. Data and findings consistent | Was there consistency between data presented and the findings? | Yes |
| 31. Clarity of major themes | Were major themes clearly presented? | Yes |
| 32. Clarity of minor themes | Is there a description of diverse cases or minor themes? | Yes, all categories described and positioned hierarchically |
